# Mesenchymal Stem Cells From Different Sources in Meniscus Repair and Regeneration

**DOI:** 10.3389/fbioe.2022.796367

**Published:** 2022-04-27

**Authors:** Guocheng Ding, Jianing Du, Xiaoqing Hu, Yingfang Ao

**Affiliations:** ^1^ Institute of Sports Medicine, Peking University Third Hospital, Beijing, China; ^2^ School of Basic Medical Sciences, Peking University, Beijing, China

**Keywords:** meniscus, stem cell, tissue engineering, meniscal regeneration, meniscal repair

## Abstract

Meniscus damage is a common trauma that often arises from sports injuries or menisci tissue degeneration. Current treatment methods focus on the repair, replacement, and regeneration of the meniscus to restore its original function. The advance of tissue engineering provides a novel approach to restore the unique structure of the meniscus. Recently, mesenchymal stem cells found in tissues including bone marrow, peripheral blood, fat, and articular cavity synovium have shown specific advantages in meniscus repair. Although various studies explore the use of stem cells in repairing meniscal injuries from different sources and demonstrate their potential for chondrogenic differentiation, their meniscal cartilage-forming properties are yet to be systematically compared. Therefore, this review aims to summarize and compare different sources of mesenchymal stem cells for meniscal repair and regeneration.

## Introduction

Over the years impaired menisci were considered functionless and were often treated via entire or partial meniscectomy. Meniscus is a crucial structure within the femorotibial joint that shoulders the responsibilities of shock absorption, load sharing, and knee stability maintenance (Fox et al., 2012). Both meniscus injury and meniscectomy can contribute to a significant change in the mechanical distribution of knee joint, which leads to secondary osteoarthritis (OA). This study aims to review the frontier of tissue-engineered stem cell therapies of menisci injury. This study aims to review the status of tissue-engineered stem cell therapies, dividing them into mesenchymal stem cell (MSC) therapy and co-culture treatments, as the current meniscal repair preferred management option requires improvement.

Situated between the femur and tibia, menisci are crescent-shaped, fibrocartilaginous structures with heterogeneous cells. The meniscus has unique mechanical properties, including Young’s modulus, tensile modulus, and compression modulus. Not only can the meniscus absorb shock, decelerate the relative speed, and perfect the morphology matching but also can lubricate the joint and assist in maintaining the anterior and posterior orientation and rotational stability of the knee joint. Physical activity is closely relevant to collagen fibers that provide meniscal structure. Meniscus fibers are typically oriented based on the three layers of the meniscus: fibers comprise three different forms: grid-like form on the surface; radiant form in the middle layer; circumferential circular form for the inner (Vonk et al., 2010).

The outer one-third of the meniscus, consisting of elongated fibroblast-like cells, is known as the “red zone”. The copious supply of blood contributes to its strong self-healing abilities. Meanwhile, the inner two-thirds, consisting of round chondrocyte-like cells, is known as the “white zone”. The avascular nature contributes to its weak healing abilities. Partial or total meniscectomy is the preferred form of treatment for a meniscus injury, which leads to the progressive degeneration of articular cartilage and eventually resulted in OA. Alternatively, a torn meniscus can be clinically sutured but will not heal due to the avascular structure of the inner meniscus. While meniscal allograft transplantation has been studied to improve joint function and alleviate joint pain and swelling. it remains a major concern due to insufficient donor sources, graft biocompatibility, immune rejection, potential infection, and uncertain long-term follow-up efficacy. For example, the use of inappropriate tissue or graft as a meniscus replacement might aggravate the injury and do more harm than no treatment.

In orthopedics or sports medicine, there is an increasing demand for minimally invasive treatments. Cell-culture techniques have heralded the age of regenerative medicine and highlighted the potential of new techniques to culture stem cells (Niu et al., 2016). It has been reported that chondrocytes can adhere and bond to the articular cartilage matrix, confirming the feasibility of chondrocyte-seeded cartilaginous scaffolds for repairing meniscus (Peretti et al., 2001). Given the complex phenotype of meniscus tissue, the use of single-resource MSC cultures may be inadequate in imitating the native meniscus well. Researchers have explored the co-culturing of different cell sources to overcome the shortcomings of monoculture. Gunja and Athanasiou (2009) innovatively co-cultured passaged meniscus cells (MCs) other than those primarily generated on poly-L-lactic acid (PLLA) scaffolds. Since the second generation of MCs has been proven to produce an extracellular matrix (ECM), their application was aimed to solve the limitation of primary MC numbers, and thereby laying a foundation for subsequent stem cell culture research. The co-culture of articular chondrocytes (ACs) and fibrochondrocytes (FCs) has been effective (Tarafder et al., 2019), but the quantity of cells required is big while the cells available for tissue engineering are limited. Therefore, the use of stem cell amplification and differentiation may hold the key to obtain enough cells for treatment.

## Stem Cell Therapies

The growth of regenerative medicine involving stem cells, including meniscal regeneration, has been witnessed over the past decade. With multidirectional differentiation being the prominent characteristic, stem cells such as bone marrow mesenchymal stem cells (BMSCs) and adipose-derived stem cells (ASCs) have become the focus of trials on meniscus injury repair in recent years, owing to their wide distribution and species diversity (Niu et al., 2016). Immune privileged status and paracrine activity of the stem cells can upscale the biomimetic properties of synthetic scaffolds employed in meniscus damage repair. Apart from BMSCs and ASCs, other sources include mesenchymal stem cells derived from the synovium and meniscus, thus creating more possibilities.

It is important to note that the term “mesenchymal stem cell” or “mesenchymal stromal cell” is broadly used and has come to mean any adherent fibroblastic population of cells, and due to their multipotency, it was speculated that these cells could form other mesodermal connective tissues outside their lineage (De Luca et al., 2019). However, neither of these terms is scientifically accurate. Mesenchyme is an embryonic connective tissue that forms not only connective tissues, but also blood and blood vessels (primarily mesodermal in origin). There is no common embryonic source for skeletal tissues, and there is no reason to believe that there would be one in the post-natal organism (Arora and Robey, 2022). In addition, BMSC populations contain a sub-population of bona fide skeletal stem cells (SSCs) in association with other cells of the marrow stroma and only the SSCs are truly multipotent. Therefore, some scientists like Caplan (2017) have proposed that MSC’s name should be changed. However, most experimental studies in the last 2 decades have habitually adopted the term “mesenchymal stem (stromal) cell”, and although the concept is not rigorous, the research conclusions are still valid. Since there is no updated naming standard, this review still uses the term MSCs for now.

BMSCs showed different differentiation characteristics when implanted in different regions of the acellular meniscus matrix (Shimomura et al., 2017). Recently, ASCs is becoming an emerging topic and only second to BMSCs from a clinical standpoint. Apart from its greater proliferation and cartilage differentiation potential, surgery to obtain ASCs from the knee joint cavity is more convenient and less invasive than that to obtain BMSCs. Synovial mesenchymal stem cells (SMSCs) as meniscus tissue engineering materials were reported to show greater chondrogenic potential and lesser osteogenic potential than BMSCs (De Bari et al., 2008). Huang et al. (2016) found a unique cell population, meniscus-derived mesenchymal stem cells (MMSCs), in rabbit meniscus that had universal stem cell characteristics and chondrocyte differentiation tendencies. The peripheral vascular area (red zone) of the meniscus was also reported to be rich in stem cells with differentiation potential, supporting the use of MMSC for meniscus repair (Osawa et al., 2013). The jury is still out on the absolute best source of stem cells since all sources have their strengths, weaknesses, and differentiation capacities. Table 1 summarizes the differentiation characteristics, clinical advantages and disadvantages of mesenchymal stem cells from different sources covered in this paper. The features and comparison will be discussed in more detail below.

**TABLE 1:** Summary table showing differentiation capacities as well as advantages and disadvantages of mesenchymal stem cells from different sources.

MSC Source	Differentiation Characteristics	Clinical Advantages	Clinical Disadvantages
Bone marrow	Strong chondrogenic and osteogenic differentiation	Aspiration can be done under local anesthesia	Invasive; Painful; Low yield
Synovium	Less cell hypertrophy differentiation than BMSCs	Abundant in the articular cavity	Staged surgery, cells require expansion
		Less painful	
		Minimally invasive	
		Minimal tissue requirement	
Adipose	Inferior in chondrogenic and osteogenic differentiation capacities	Less painful than marrow aspiration; high yield	Local anesthesia toxic to ASCs therefore harvest
	Superior in adipogenic differentiation		preferable under GA
Meniscus	Chondrogenic differentiation	Easy to controlled by signaling pathways	Pain
	Less CD34 expression than in BMSCs; less osteogenic differentiation than BMSCs		Irreversible damage to donors
	—		Cells require expansion
Peripheral blood	Chondrogenic differentiation capacity is poor	Abundant and less painful	Low yield
Menstruation	Chondrogenic properties have not been explored	Painless	Low yield
		The most accessible sample source	Fixed time available
Induced pluripotent stem cells	Pluripotency; differentiation into any somatic cell type under appropriate conditions	—	Expensive
			Time-consuming
Tendon	Fibrochondrogenic differentiation	Excellent viability, distribution and proliferation	Low yield and complicated operation
			Invasive
Cartilage	Less cell hypertrophy differentiation than BMSCs	Easy to controlled by signaling pathways	Low yield and invasive

**FIGURE 1:**
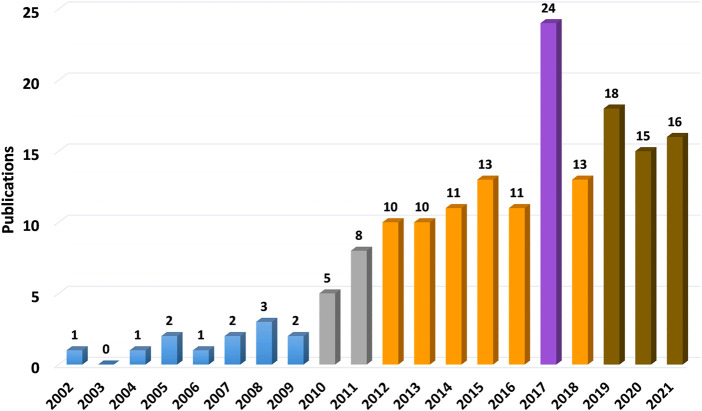
Stem cells employed in meniscal repair studies. Stem cell research for meniscus repair shows a boom in the past decade, with a peak in 2017 for stem cell research of all kinds and then declining slightly. In this column diagram, different colors correspondingly represent article numbers of that year. Blue ones are less than 5 (5 is not included); gray ones are from 5 to 9; orange ones are from 10 to 14; brown ones are from 15 to 19; purple one is more than 20.

**FIGURE 2:**
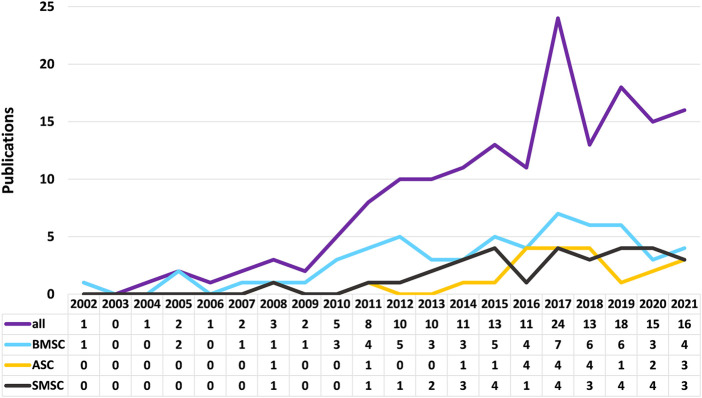
Trends in the number of stem cell studies on bone marrow mesenchymal stem cells, adipose-derived stem cells, and synovial mesenchymal stem cells.

This article aims to comprehensively review the status of mesenchymal stem cells for meniscal repair and regeneration, including several common and uncommon cell sources, in animal and human experiments. The literature search for this review was conducted in July 2021. The Web of Science database was used with the following MeSH terms: TI = menisc* AND TS = (repair OR regeneration) AND AB = (mesenchymal stem cell * OR stem cell *) AND PY = (2000–2021). TI represents Title; TS represents Topic; PY represents Year Published; AB represents Abstract. A total of 166 articles were found **(** Figure 1). When comparing on a year-to-year basis, stem cell research for meniscus repair shows a boom in the past decade, with a peak in 2017 for stem cell research of all kinds and then declining slightly. Among all the articles, the top three kinds of stem cells with the largest number are BMSCs, synovial mesenchymal stem cells (SMSCs), and ASCs. BMSC was the first stem cell type to be studied, and in line with the overall trend, with a peak in research in 2017. Studies on SMSC and ASC started late, increased rapidly in the last 5 years, and continue to rise (Figure 2). All data were extracted and reviewed from article texts, tables, and figures.

### Bone Marrow Mesenchymal Stem Cells

#### Bone Marrow Mesenchymal Stem Cell Therapies

**FIGURE 3:**
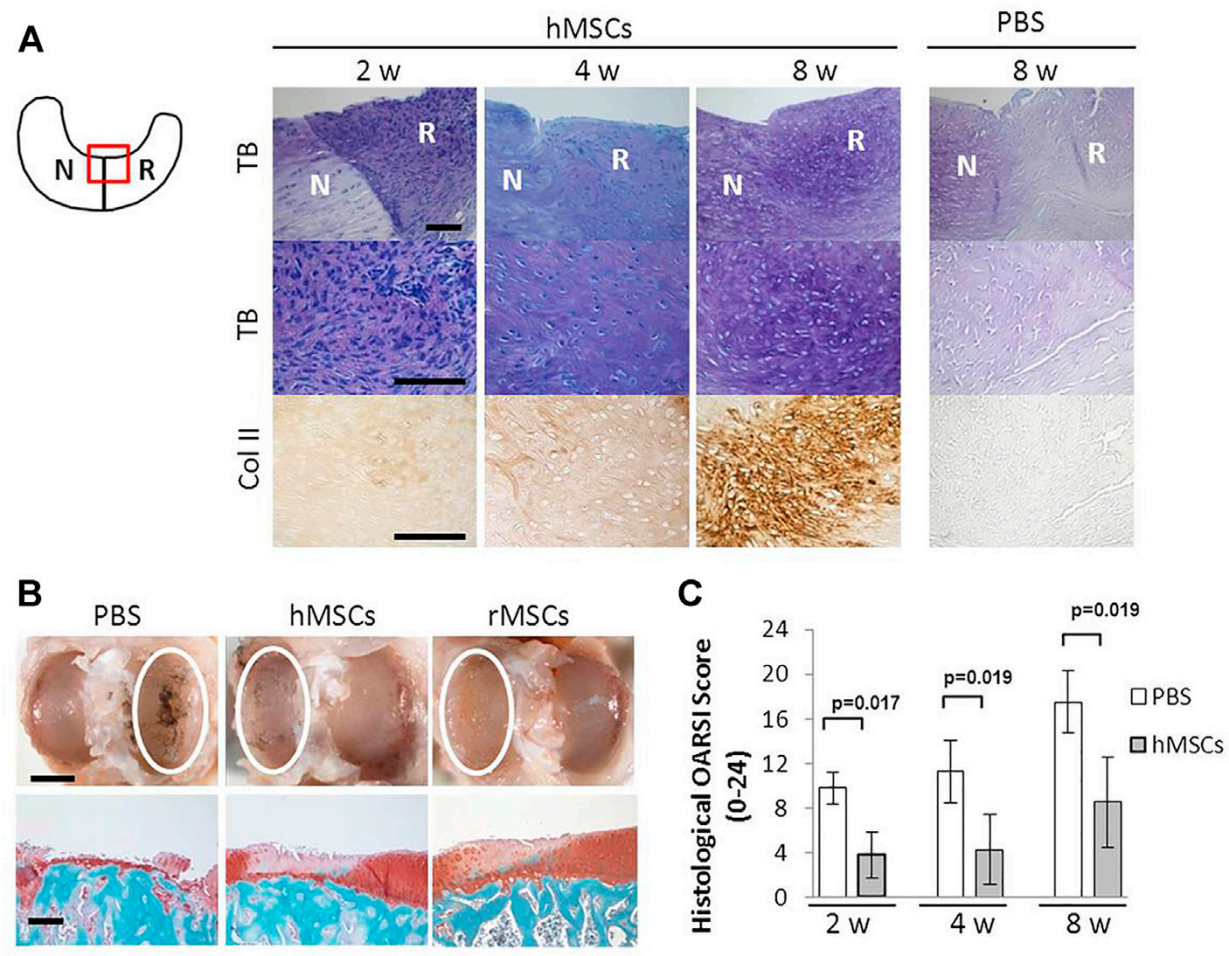
Intra-articular injection of hMSC promoted regeneration of rat meniscus **(A)** Representative sections of the meniscus stained with Toluidine blue (top and middle), and immunostained for type II collagen (bottom) after PBS or hMSCs injection. The staining in the PBS-treated sample was less with Toluidine blue and the antibody for type II collagen. The schema of the meniscus on the left is shown for orientation. Scale bar, 100 μm. **(B)** Representative gross photographs (top) and sections (bottom) of the joint surface of the tibia at 8 weeks. The cartilage was stained with India ink to identify fibrillation and erosion. The white circle indicates the medial tibial plateau. The tibia was sectioned coronally and stained with safranin-O and fast green to identify cartilage (red). Scale bars, 2 mm (top) or 200 μm (bottom). **(C)** Quantification of histological analysis using the OARSI cartilage osteoarthritis histopathology grading system. Values are mean with lower and upper limit of 95% CI; n = 5 for each group (Mann–Whitney U test). Abbreviations: hMSC, human mesenchymal stem cell; rMSC, rat mesenchymal stem cell; N, native meniscus; R, regenerated meniscus; TB, Toluidine blue; Col II, type II collagen. Adapted with permission from Horie et al. (2012).

From our literature study on stem cell trials regarding meniscal defect repairs, BMSCs account for most of the trials. Studies using small and large animal models showed that direct intra-articular injected BMSC shad a good effect on meniscus repair, especially in the avascular zone. Figure 3 shows the representative results of promoted regeneration of rat meniscus after intra-articular injection of human MSCs. It also provided cartilage protection (Horie et al., 2012; Caminal et al., 2014; Yuan et al., 2017). The ease and safety of cell injection promoted the implement of human clinical trials (Centeno et al., 2008; Vangsness et al., 2014), where human BMSCs (hBMSCs) extracted from the iliac bone marrow were injected percutaneously into the knee joint of patients, which showed increased cartilage growth, reduced pain, and increased joint flexibility in most of the patients. These studies revealed a modality of combining meniscal allograft transplantation and stem cell injection.

Due to the frozen storage of human allograft specimens, inadequate cellular repopulation may impair graft viability. Struijk et al. (2021) injected doses of BMSCs (>0.1 million) in meniscus allograft tissue, which resulted in active cell proliferation, migration, and robust cell survival. Additionally, allogeneic hBMSC injection showed graft versus host disease (GvHD) inhibition and strong anti-inflammatory and regenerative capacity, which could be attributed to the ability of BMSCs to produce and release bioactive factors. Despite these applications, when using single-resource MSC suspension injection, only a small percentage of functional cells remain at the target site. To solve this issue, the use of mesenchymal stem cell sheets was introduced by Qi et al. (2016a). The mesenchymal stem cell sheet can decrease cell loss obviously and, since the visible sheet is easy to place around the meniscal defect, it can directly fill the space caused by meniscectomy.

Direct injection of cells *in vivo* can promote cellular repair, but it cannot meet the mechanical requirements of the meniscus (Desando et al., 2016). This leads to the emergence of scaffold-based cell therapies. As early as in 2005, implantation of rat BMSCs together with acellular meniscus scaffolds was reported, showing comparable results to native meniscus chondrocytes and a better morphology than a deep-frozen allogeneic cell-free implant (Yamasaki et al., 2005). Thereafter, animal studies were conducted to implant BMSCs on artificial synthetic or modified natural scaffolds, and different kinds of cytokines or small molecule compounds added in trials performed well; mechanical stimulation also revealed good experimental results (Yuan et al., 2017; Stuckensen et al., 2018; Liu et al., 2019; Zhao et al., 2020).

In addition to the use of specific stem cell species, other factors also influence meniscus repair and regeneration. A scaffold provides a microenvironment for stem cell attachment and cell growth that helps to repair the damaged or torn part of the meniscus. The challenge in scaffold optimization is to achieve a delicate balance between mechanical strength and biological activity. The mechanical and degradation properties of synthetic or natural materials used for tissue engineering meniscus scaffolds should be considered to ensure immune compatibility. Moreover, it is necessary to ensure that the scaffold can provide recruitment, proliferation and secretion of ECM for tissue engineered cells. Ying et al. (2018) combined BMSC with silk protein to repair rabbit meniscus; Achatz et al. (2016) planted hBMSC on a polyurethane scaffold for *in vitro* meniscus repair; Filardo et al. (2019) planted hBMSCs on a three dimensional printed collagen meniscus scaffold and characterized cell activity and distribution. Whitehouse et al. (2017) applied an autologous BMSC collagen scaffold to repair five rows of human meniscus injuries, and three cases had good results after 2 years of the surgery while two cases required meniscectomy. Other studies also report the implantation of BMSCs on different artificial polymer scaffolds, and various *in vitro* or *in vivo* experiments have verified that BMSCs are equipped to promote meniscus regeneration and articular cartilage protection (Zhang et al., 2017; Zhong et al., 2020).

However, stem cells’ infiltration into these scaffolds is low, owing to their dense ECM structure and mismatched size issues in the joint. Hence, chemotactic agents such as growth factors have been explored to stimulate cell migration and infiltration, and thereby enhancing integration into the scaffold. Growth factors such as connective tissue growth factor (CTGF) and transforming growth factor-beta 3 (TGF-β3) act on target cells to stimulate cellular growth, proliferation, healing, and differentiation (Tarafder et al., 2019). Hu et al. (2019) investigated the inductive effects of silver nanoparticles on osteogenic differentiation and proliferation using MSCs, and the advancement of meniscus injury healing. However, Zellner et al. (2014) evaluated the effects of growth factors like platelet-rich plasma (PRP) or bone morphogenic protein 7 (BMP7) on avascular meniscal defect regeneration, which failed to significantly improve meniscus healing in the avascular zone in a rabbit model after 3 months. Thus, several certain chemotactic agents may as well make a difference to the scaffold-based tissue engineering in meniscus regeneration and more candidates await to be investigated in the future.

#### Bone Marrow Mesenchymal Stem Cells Co-cultured With Other Cells

As BMSC numbers isolated from bone marrow are still limited, most studies on cartilage regeneration use culture-amplified cells. Recently, increasing studies have focused on the co-culture of BMSCs with MCs or FCs, and both ECM synthesis and related gene expression are superior to those of the monoculture group, suggesting that BMSC differentiation phenotype is affected by FC or MC (Cui et al., 2012; McCorry et al., 2016; Zellner et al., 2017). Studies report that the co-culture of primary MCs and MSCs with direct cell-cell contact enhanced extra chondral matrix generation either under normoxia or hypoxia (Matthies et al., 2013). Compared with FCs’ monoculture, the co-cultured group yielded more glycosaminoglycans (GAGs) and collagen production. This could be due to MSCs may increase matrix production but has less capacity on forming fibrous organization as compared to FCs (McCorry and Bonassar, 2017). Hence, cell co-culture can be optimized in tissue engineering with balanced MSC synthesis characteristics and FC matrix remodeling ability.

Given the unique advantages of cell co-culture, a plural of studies have been performed to investigate the optimal ratio between cultured MCs and BMSCs. Cui et al. (2012) reported that MC and MSC co-cultured at a ratio of 75:25 yielded the highest collagen I and GAG and expressed the lowest hypertrophic differentiation genes. McCorry et al. (2016) reported the highest GAG retention in the 50:50 co-culture ratio, and the highest polymerization modulus was obtained both at 100:0 (MSC single culture) and 50:50 co-culture ratios. Recently, Hagmeijer et al. (2019) reported that the best meniscus structure was obtained when MC and MSC were co-cultured at a ratio of 20:80. While most studies focused on co-culturing BMSCs and MCs, (Saliken et al., 2012) compared cells from the inner (I) and outer (O) meniscus respectively. Their results showed that although primary MCOs or MCIs co-cultured with BMSCs had similar synergistic effects that increased matrix formation, MCOs were more advantageous in inhibiting the hypertrophic differentiation of MSCs. The reason for this difference was unclear though. These suggest that MCs from the outer vascular regions of the meniscus can be supplemented with MSCs to engineer functional grafts for inner avascular meniscus reconstruction. However, these findings contrasted to those of their previous studies (Adesida et al., 2007). Donor variability (age, gender, and so on) is one plausible reason and further study deserves to be carried out.

**FIGURE 4:**
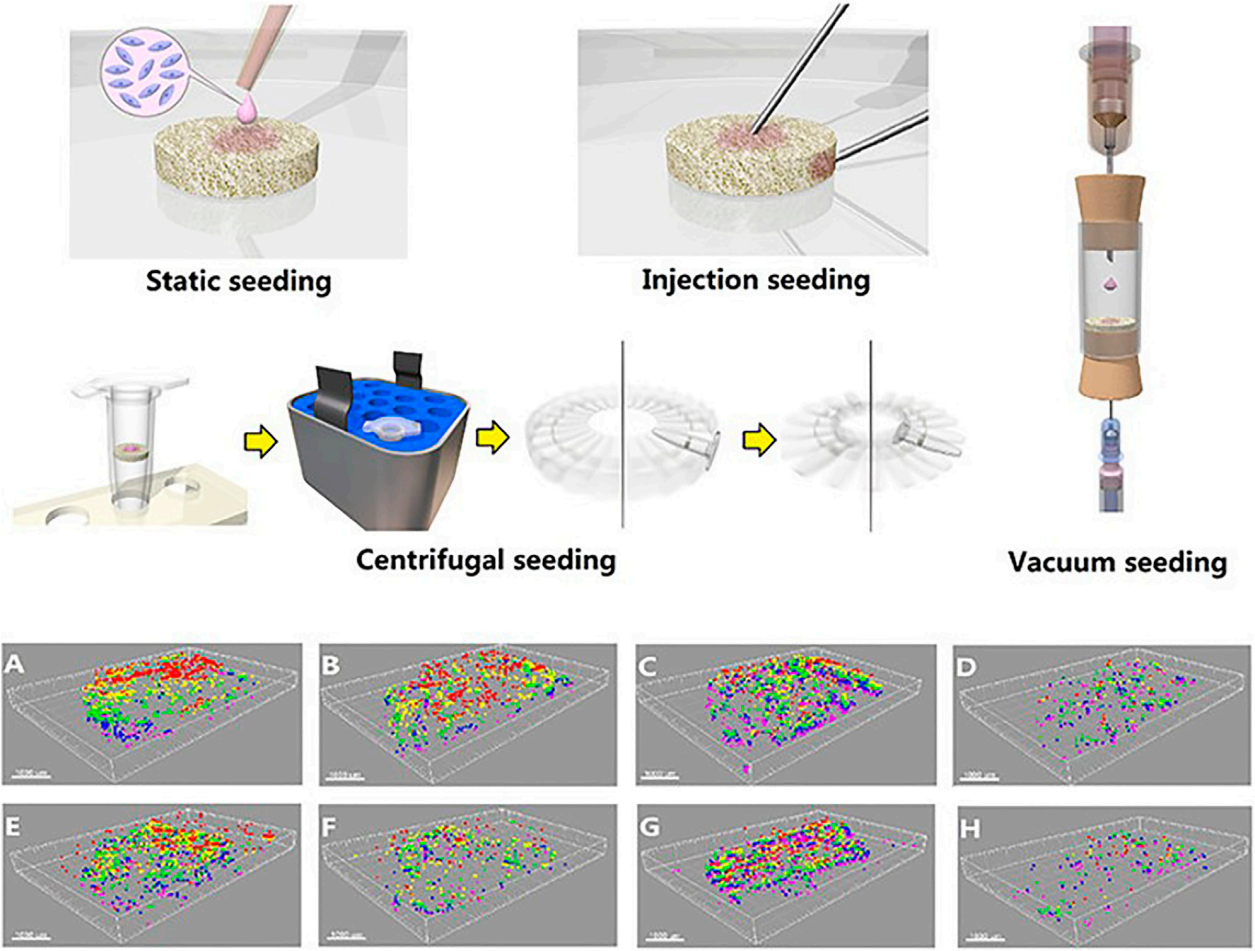
3D images of the cells distribution in scaffolds using different cell seeding methods. **(A)**–**(D)** Seeded with MSCs; **(E)**–**(H)** seeded with MFCs. **(A)**,**(E)** Surface static seeding; **(B)**,**(F)** injection seeding; **(C)**,**(G)** centrifugal seeding; **(D)**,**(H)** vacuum seeding. (Red, 0–100 μm; yellow, 100–200 μm; green, 200–300 μm; blue, 300–400 μm; purple, 400–500 μm). Adapted with permission from Zhang et al. (2015).

Tissue engineering provides a possible solution to the problem of regeneration and replacement after meniscectomy. However, the uneven distribution of seed cells on scaffolds and the decreased proliferation ability hinder the application of tissue-engineered meniscus as a new generation of meniscus graft. The centrifugal seeding method, a simple and cost-effective cell-seeding protocol for tissue-engineered meniscus, was reported to significantly improve FCs or BMSCs distribution and proliferation on the demineralized cancellous bone scaffolds (Zhang et al., 2015). Static seeding, injection seeding, centrifugal seeding, and vacuum seeding methods were used to seed the meniscal FCs and mesenchymal stem cells to scaffolds. Combined with the reconstructed three-dimensional image, the distribution of seeded cells was investigated (Figure 4). By using a scaffold composed of Col I gel on the small intestine submucosa combined with a co-culture of MCs and BMSCs, a construct similar to the native meniscus tissue in its GAG/DNA expression in addition to Col I, Col II, and aggrecan production was observed (Kremer et al., 2017). It should be noted that growth factors also have potential to promote meniscus recovery. However, the current research findings are yet inadequate to draw a solid conclusion.

A major limitation of cell engineering therapy is that the repaired cartilage tissue may disappears from the damaged area over time and requires a follow-up surgery. For example, Somoza et al. (2014) reported that neither exogenous nor endogenous BMSCs could promote long-lasting phenotypic correction (i.e., anatomical site-specific) and articular cartilage regeneration. Therefore, a better source of chondrocytes and a better understanding of the key environmental cues for articular chondrocyte development are expected. Other limitation of current preclinical study of cell engineering therapy includes the knee joint of employed small animal models (e.g. rabbits) has great difference from human knee joint. Studies using larger animal models are needed to establish a more comprehensive understanding of the underlying mechanism of cell engineering that leads to its clinical application.

### Synovial Mesenchymal Stem Cells

#### Synovial Mesenchymal Stem Cell Therapies

Matsukura et al. (2014) studied the knee fluid of MSCs after trauma. Their results suggested that SMSCs were the main stem cells mobilized after a knee injury and could be a critical cell source for meniscus repair. When compared with BMSCs, obtaining SMSCs is minimally invasive, has minimal tissue requirement, and can be harvested during a simple arthroscopic procedure (Jacob et al., 2019). Still, due to the limited number of cells in the synovial fluid, staged surgery and expansion of cells are required, which is a potential disadvantage of the clinical applications. Taking this disadvantage into consideration, Baboolal et al. (2018) discussed the feasibility of SMSC mobilization during the knee arthroscopy process and thereby providing a theoretical basis for the future evaluation and clinical application of natural joint MSC in joint repair. Three irrigation fluid samples were collected from each patient after initial examination of the joint and synovium agitation at the beginning of knee arthroscopy. Employing this technique to mobilize cells in the synovium during arthroscopy can greatly amplify the number of functional mesenchymal stem cells. Moreover, the technique is easy to use and has no complications.

Comprehensive studies have been performed to establish the optimal differentiation conditions of SMSCs. The factors that promote SMSCs differentiation, exogenous growth factors were tested by Liang et al. (2018) on the meniscus-derived decellularized matrix with SMSCs. Their results showed an upregulation in the expression of aggrecan, Collagen I, and Collagen II. A study by Tarafder et al. (2018) reported that the controlled applications of CTGF and TGF-β3 can induce seamless healing of avascular meniscus tears by inducing the recruitment and step-wise differentiation of SMSCs. Additionally, chemical and physical conditions have also been further investigated, with promising results in PCL electrospinning technology, magnesium ions, 3D printing-based biomimetic, and composite tissue-engineered meniscus scaffold (Shimomura et al., 2019; Zhang et al., 2019; Li et al., 2020; Li et al., 2021). To ensure clinical efficacy, the proper transport of SMSCs from the processing facility to the clinic is important. Mizuno et al. (2017) verified that the viability and chondrogenic potential of SMSCs were preserved when the cells were suspended in complete human serum, and the optimal temperature was at 4°C or 13 C.

**FIGURE 5:**
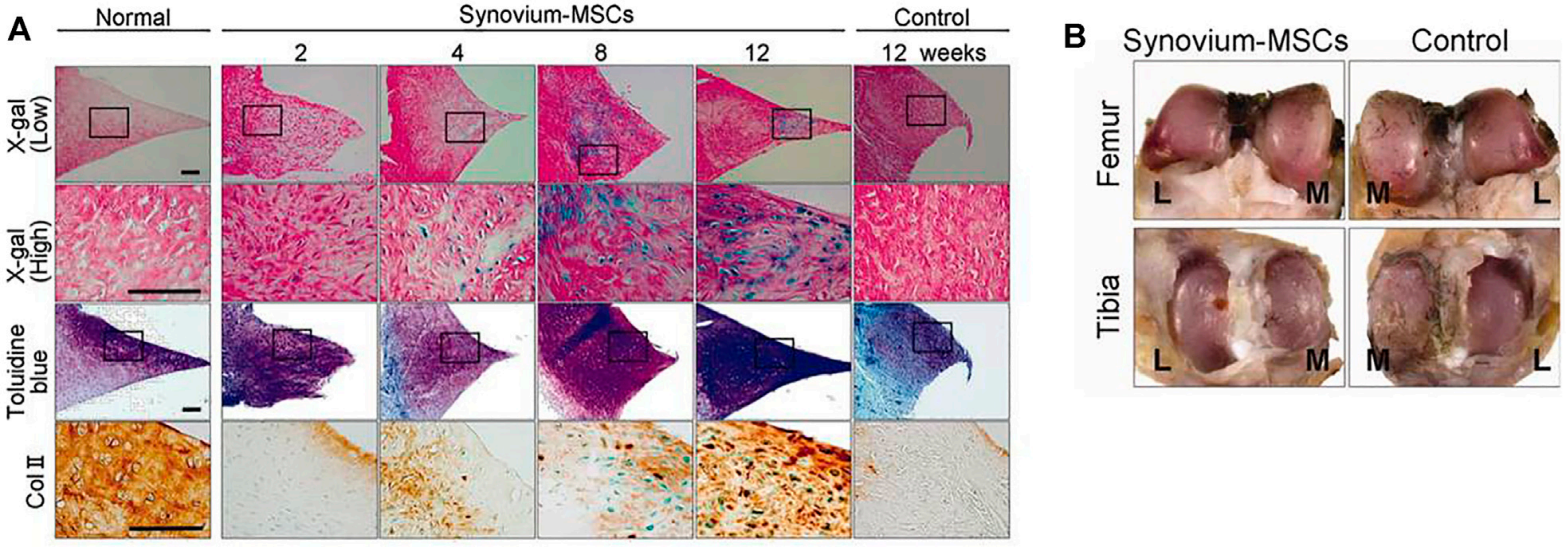
Histological and macroscopic observation of meniscal regeneration after the intra-articular injection of MSCs derived from Luc/LacZ transgenic rats. **(A)**: Representative sections of normal meniscus and regenerated tissues in the synovium-MSC injection group stained with X-gal (and eosin as counter staining), toluidine blue, and immunostained with collagen type 2. Scale bar = 100 μm. **(B)** Representative macroscopic findings of the joint surface of femur and tibia 12 weeks after the synovium-MSC group and the control group. The cartilage was stained with India ink. Abbreviations: L, lateral; M, medial; MSCs, mesenchymal stem cells. Adapted with permission from Horie et al. (2009).

SMSCs have been used in various preclinical trials to repair meniscus defects in rat, rabbit, and pig models to regenerate fibrocartilage in animal experiments can be effectively generated based on these results (Katagiri et al., 2013; Nakagawa et al., 2015; Ozeki et al., 2021). Figure 5 shows the representative results of meniscal regeneration after intra-articular injection of SMSCs derived from Luc/LacZ transgenic rats. Good proliferation and chondrogenic potential make SMSCs a potent cell source for cartilage and meniscus regeneration (Li et al., 2017; Watanabe et al., 2020). Compared to BMSCs, differentiated SMSCs showed lesser expression of collagen X, a marker of cell hypertrophy differentiation. Kondo et al. (2017) were the first to investigate autologous SMSCs in primate experiments. Their results showed that it could promote meniscus regeneration and reduce degeneration of articular cartilage.

Agreeing with preclinical findings, clinical trials that directly inject SMSCs into the knee joint to treat meniscus defects and OA showed a restoration of the meniscus function and cartilage protection (Saulnier et al., 2015; Suzuki et al., 2020; Sekiya et al., 2021). SMSCs combined with the surgical suture repair of a complex degenerative tear of the medial meniscus could not only improve the symptoms of patients but also had no obvious adverse reactions (Sekiya et al., 2019). For now, SMSCs have shown promising prospects, whether being injected directly into the joint cavity or cultured on a scaffold. More experiments have yet to be repeated to facilitate the clinical application of SMSCs.

#### Synovial Mesenchymal Stem Cells Co-cultured With Other Cells

To address the shortage of autologous MCs, some researchers designed a co-culture system of SMSCs with MCs to produce large amounts of cells while maintaining characteristics of MCs. By co-culturing SMSCs with MCs *in vitro*, Song et al. (2015) proved that SMSCs could support the proliferation and collagen synthesis of FCs by secreting FGF1. Before that, Tan et al. (2010) had co-cultured MCs and SMSCs on small intestine submucosa (SIS) to establish an *in vitro* engineered meniscus construction method. They mixed approximately 0.9 million cells with fibrin gel, which were seeded onto lyophilized SIS discs and then incubated in a serum-free medium supplemented with 10 ng/ml TGF-β1 and 500 ng/ml insulin-like growth factor I for 1 month. The results showed that the co-culture of SMSCs and MCs constructs had greater cell survival and chondrogenic differentiation phenotypes; higher glycosaminoglycan, collagen II, and Sox 9 but lower collagen I, which lead to an increase in equilibrium modulus. This preliminary study confirms the advantages of MCs and SMSCs co-cultivation in meniscus tissue engineering and regeneration.

The co-culture of MCs and SMSCs has shown its advantage over the monoculture of each population; however, the optimal ratio to be used remains to be solved. Different ratios of SMSCs and MCs at 3:1, 1:1, and 1:3 were tested by Xie et al. (2018). The proliferation and differentiation abilities were compared. The co-culture of SMSCs/MCs at the ratio of 1:3 showed better results than the control groups (monoculture of SMSCs or MCs) or those at other ratios, indicating the most secretion of sGAG, a marker of chondrogenic differentiation. SMSCs are abundant in the articular cavity, easy to obtain, and have multi-directional differentiation potential. This MCs and SMSCs co-culture system may be a promising strategy for meniscus repair with tissue engineering. A limitation of this approach is that the localization of SMSCs has not been fully studied, and its specific markers and MSCs characteristics in each region of the synovium need further study.

### Adipose-Derived Stem Cells

#### Adipose-Derived Stem CellTherapies

Recently, ASCs have gained popularity due to the ease of procurement via liposuction. Hoffa’s fat pad is an adipose body in the knee and being anatomically close to the meniscus. It is an intracapsular extra synovial source of ASCs for meniscus tissue engineering (Abpeikar et al., 2021). Given the less painful harvest process than marrow aspiration and higher yield, ASCs are a good alternative to BMSCs in equine meniscus repair (González-Fernández et al., 2016). Baek et al. (2018) employed an electrospinning collagen scaffold for *in vitro* culturing adult MMSCs, BMSCs, SMSCs, and ASCs derived from the infrapatellar fat pad (IPFP). Their results demonstrated that meniscal-like tissue produced by ASCs had higher meniscus gene expression, better mechanical properties, and better cell distribution.

To evaluate the practical applicability of ASCs, autologous and allograft ASCs were tested in a rabbit meniscus longitudinal tear injury model. ASCs allograft was placed either in the lesion and filling the defect area as cylindrical plugs implanted with collagen scaffolds, or directly injected into the joint cavity. Both methods enhanced suture repair and suppressed inflammation (Qi et al., 2016b; Toratani et al., 2017; Takata et al., 2020). In a larger animal model, Rothrauff et al. (2019) applied autologous IPFP-derived ASCs to repair radial meniscus tears of goats. They reported enhanced repair with a photo-cross-linked hydrogel containing TGF-β. Autologous IPFP-derived ASCs could improve meniscus healing and reduce deterioration into OA. Cengiz et al. (2020) planted Hoffa’s fat pad-derived ASCs that were isolated from human tissues on PCL silk fibroin protein complex scaffolds. *In vitro* culture, showed a valuable regenerative potential of ASCs. However, the potential of hASCs derived from Hoffa’s fat pad to differentiate into MCs and the similarity of the new tissue to native MCs and ECM remains to be studied.

Early reports showed ASCs were inferior to SMSCs in terms of their chondrogenic and osteogenic differentiation capacities (Sakaguchi et al., 2005), leading to a water-down curative effect compared to SMSCs’ of the same cell quantity. Despite these disadvantages, ASCs has its unique advantages. Sasaki et al. (2018) isolated hASCs and planted them in a photo-cross-linked methacrylic gelatin hydrogel to form a 3D structure and added TGF-β3 for *in vitro* culture. Their results showed satisfactory healing abilities, which were evaluated at 4 and 8 weeks of culture using histology, immunofluorescence staining, and mechanical tests. Clinical trials using intra-articular injected ASCs reported a satisfactory effect on meniscus repair and cartilage protection. Efforts were also made to overcome the shortage of ASCs such as cell loss and low survival rate. Nordberg et al. (2016) showed acupuncture perforation promoted the infiltration of human ASCs (hASCs) in the meniscus, which may improve the efficacy of meniscus transplantation. Cell sheets were also used in ASCs to increase the number of effective cells and fill the wound space (Takata et al., 2020). Moreover, mechanical stimulation can increase the expression of meniscus-related RNA level in ASCs and promote cell differentiation (Meier et al., 2016).

#### Adipose-Derived Stem Cells Co-cultured With Other Cells

**FIGURE 6:**
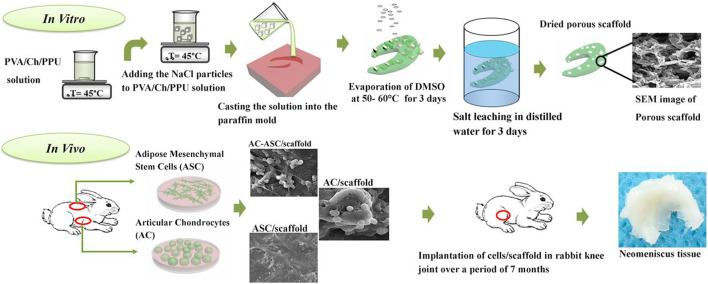
Graphical abstract of the scaffolds fabrication, cell culture and implantation of the tissue-engineered meniscus constructs in rabbit knee joint. Adapted with permission from Moradi et al. (2017).

There are little reports on the co-culture of ASCs with other somatic cells and the results are not encouraging. Moradi et al. (2017) implanted ACs and ASCs into scaffolds in different ratios and found that the AC/scaffold group was the best, followed by the AC-ASC/scaffold and ASC/scaffold groups. Additionally, the AC/scaffold group had the best articular cartilage preservation and highest histological score, suggesting that the PVA/Ch/PPU (Ch4) scaffold implanted with AC alone could successfully regenerate the torn meniscus, with ASC providing no significant contribution to the healing process. Despite these results, the applications of ASCs in meniscus regeneration cannot be dismissed. Although animal models and specific materials and methods are different among various research, the experimental ideas are basically the same. Figure 6 shows the flow path of this study, which can be used as a typical example for experimental design of scaffold-based stem cell co-culture.

### Meniscus-Derived Mesenchymal Stem Cells

The meniscus itself is rich in stem cells during its early development (Gamer et al., 2017). The developmental changes in cell morphology and structure of MMSCs are substantial (He et al., 2019), reflecting the complexity of MMSCs differentiation in different location and developmental stages. Thus, MMSCs are a potent cell source for meniscus tissue engineering. These meniscus progenitor cells are multipotent, exhibiting migratory activity, and are likely to be controlled by canonical TGF-β signaling that leads to an increase in SOX9 and a decrease in RUNX2 (Muhammad et al., 2014). Therefore, studies relevant to signaling pathways promote the development of novel cellular, biological, and regenerative therapies for meniscus repair.

To evaluate whether or not MMSCs are advantageous to other stem cell populations, Huang et al. (2016) compared MMSCs, BMSCs, and FCs in rabbit meniscus repair. The results showed that MMSCs had smaller cell bodies and larger nuclei, with common stem cell characteristics and chondrocyte differentiation abilities. Additionally, MMSCs not only have smaller colonies and slower growth rates but also have lesser CD34 expression than that in BMSCs. Finally, MMSCs always exhibited a clear tendency of chondrogenic differentiation either *in vivo* or *in vitro*, while BMSCs showed higher osteogenic potential. MMSCs were then injected into the joints of animals with meniscus defects to test their capacity in tissue regeneration, resulting in promoted cell migration (Shen et al., 2013; Shen et al., 2014). Nevertheless, (Zellner et al., 2017) used collagen hydrogel scaffold to compare rabbit autologous BMSCs and MMSCs *in vivo* and found that BMSCs had better clinical applications. Whether BMSCs or MMSCs are more suitable for regenerative medicine is yet to be determined using more multidimensional and repeated experiments *in vivo* or *in vitro*.

### Mesenchymal Stem Cells From Other Sources

Since the transplantation of meniscal allograft or artificial menisci is limited by graft sources and adverse events, substitution for meniscus reconstruction still needs to be explored. Apart from the common types of stem cells discussed above, there are other innovative sources of stem cells that have been studied in tissue engineering. Hoben et al. co-cultured human embryonic stem cells with fibroblasts and showed the production of type II collagen were increased by 9.8 times (Hoben et al., 2009). However, studies in this field are very few. Some of these stem cells have only been tested for their cartilage differentiation potential, lacking *in vivo* animal trials, and cytokine production abilities that can inhibit meniscus repair. Despite the methods used and results obtained from these studies, they help to advance regenerative medicine in meniscus repair. (Table 2).

**TABLE 2:** Various mesenchymal stem cell sources used for meniscus repair.

Hoberg et al. (2009)	Jayasuriya et al. (2019)	Hoben et al. (2009)	Koh et al. (2017)	Olivos-Meza et al. (2019)	Li et al. (2017)	References
HUVECs	C-PCs	hESCs	TMSCs	PBSCs	TDSCs	Cell Source
—	1.0 × 10^5^	5.0 × 10^5^	1.0 × 10^6^	2.0 × 10^7^	2.5 × 10^5^	Number of Cells Used
—	Three-month-old Lewis rats (*Ex vivo*)	—	6 New Zealand white rabbits (3.0–3.3 kg)	Clinical human trial	15 skeletally mature Japanese big-ear rabbits (3.0–3.5 kg)	Animal Model
Inner 2/3 of the menisci ft were minced	Radial tear in inner anterior horn	—	Meniscal defect	Partial irreparable meniscal injury or partial (or subtotal) meniscectomy	Tendon tissue defect	Meniscus injury model
Detection and quantification of cDNA by PCR	mRNA expression analysis	Immunostaining	Mechanical testing	Lysholm Scale	Macroscopy; Histology; Histomorphometry; Biomechanics	Evaluations
ELISA	Immunostaining	Histology	Immunostaining	T2 Mapping		
	Cell surface marker	Quantitative biochemistry	Real-time qPCR	—		
	Western blot	Flow cytometry	Biochemistry	—		
	—	—	Histology	—		
2 weeks: higher expression of endostatin and decreased proliferation rate of endothelial cells	3,5,10,17 and 20days: promoted FC proliferation and native tissue integration; SDF-1/CXCR4 axis is required to successfully fill meniscus tissue tears	3 weeks: The combination of growth factors BMP-4 and TGF-β3 with coculture of FC showed the best regeneration capacity	10 weeks: conditioned medium (CM)-expanded cells treated with TGF-β3 apparently promotes meniscus regeneration	12 months: fail to show any advantage in the protection of articular cartilage	24 weeks: promoted meniscal regeneration and protection of condylar cartilage	Outcome

When FCs were co-cultured with human umbilical vein colorectal cells, endostatin expression increased, endothelial cell proliferation rate was decreased, and meniscal tract repair was inhibited (Hoberg et al., 2009). Clinically, peripheral blood stem cell (PBSC) transplantation has been applied to repair meniscus. Olivos-Meza et al. (2019) conducted the first clinical application of PBSC combined with polyurethane scaffold in humans and no significant difference in articular cartilage was observed. Similarly, menstrual blood can be said to be the most accessible sample source and there is no need for complex ethical and surgical interventions. Menstrual-derived stem cells have a greater proliferation and differentiation potential than BMSCs, making them a perspective tool in clinical practice (Uzieliene et al., 2018). However, their chondrogenic properties have not been extensively explored. Clinical application of these stem cells is still to be investigated with no positive results yet.

There are several studies whose results are promising in regenerative medicine. Human pluripotent stem cells (hPSCs) can maintain pluripotency and normal karyotype during culture and can differentiate into any somatic cell type under appropriate conditions. However, due to ethical issues, PSCs used nowadays are all induced pluripotent stem cells, and thereby adding to the experimental difficulty. Semitendinosus tendon autograft was investigated for its fibrochondrogenic metaplasticity potential and chondroprotective effect (Li et al., 2017). Their experiment mainly studied two important stem cell sources: Tendon-derived stem cells and SMSCs, a combination of which exhibited excellent viability, distribution, proliferation, and fibrochondrogenic differentiation abilities in decellularized semitendinosus tendon scaffolds *in vitro*. Koh et al. (2017) investigated the effects of conditioned medium from meniscal fibrochondrocytes and TGF-beta 3 on tonsil-derived mesenchymal stem cells for meniscus tissue engineering *in vivo*, highlighting a novel stem cell commitment strategy combined with biomaterial designs. Jayasuriya et al. (2019) hypothesized that chondroprogenitor cells (C-PCs) from healthy human cartilage are better than BMSCs due to their ability to resist cell hypertrophy and to mobilize in response to chemokine signaling.

## Discussion

Studies have confirmed that mesenchymal stem cells can be isolated from various intra-articular tissues, including meniscus and ligaments, and that the gene expression profiles of intra-articular tissue mesenchymal stem cells are similar to each other: high expression of proline arginine-rich and leucine-rich repeat protein (Segawa et al., 2009). Stem cells in other well-differentiated tissues have also been explored for their capacities to differentiate into cartilage for damaged meniscus repair. Whether it is bone marrow, adipose tissue, or synovial fluid that has been widely studied, or menstrual blood, tonsil, peripheral blood that has been reported less often, different stem cell sources present different characteristics and clinical application potential. Of note, it is undeniable that the ability of animals to repair is different from humans, and meniscus injuries in some animals could be completely healed without any treatment in due course of time. Thus, if a gene modification could lead to an animal model that has a higher resemblance to pathological changes in humans, it could become an important research direction (Dai et al., 2021). The clinical application of different stem cells for meniscal repair has been summarized in Table 3.

**TABLE 3:** Summary of clinical studies using stem cell therapies.

Ref	Cell Source	Patient Inclusion	Injury	Methods	Evaluation	Observation Time	Outcome
Centeno et al. (2008)	BMSCs	46-year old male	Degenerative menisci and OA	Percutaneous injection of 22.4 million MSCs into knees	MRI	24 weeks	Meniscus and cartilage volume increased
					Visual Analogue Score (VAS)		Modified VAS pain scores decreased
					Range of motion (ROM)		Range of motion in extension increased
Whitehouse et al. (2017)	BMSCs	4 males and 1 female, aging from 30 to 38 years	Isolated medial meniscal tears in the avascular zone with intact anterior cruciate ligaments	Autologous MSC/collagen-scaffold implantations	MRI	24 months	The implant survived in 3 cases whereas 2 cases developed recurrent symptoms at around 15 months, leading to meniscectomy
					ROM		
					Tegner-Lysholm score		
					International Knee Documentation Committee (IKDC) score		
Vangsness et al. (2014)	BMSCs	55 patients with an average age of 46 years	Partial medial meniscectomy	Group A: BMSCs (5 × 10^7^), human serum albumin, sodium hyaluronate, and plasma	VAS score	12 months	A higher proportion of those with osteoarthritic changes experienced a reduction in pain
				Group B: BMSCs (1.5 × 10^8^)	MRI		
				Group C: sodium hyaluronate	—		
Sekiya et al. (2021)	SMSCs	6 patients (5 males and 1 female) aging from 45 to 62 years	degenerative flap and radial medial meniscus tear	A suspension of SMSCs was placed onto the sutured meniscus through a needle	Tegner-Lysholm score	52 weeks	The posterior junctional zone was completely restored in 2 patients and partially restored in the other 2 patients
					Arthroscopy		Lysholm scores were significantly higher at 4 and 52 weeks
					MRI		—
Olivos-Meza et al. (2019)	PBSCs	17 patients aging from 18 to 50 years	Partial irreparable meniscal injury or partial (or subtotal) meniscectomy	2 groups: acellular polyurethane scaffold or polyurethane scaffold enriched with MSC	Lysholm Score	12 months	It failed to show any advantage in the protection of articular cartilage
					MRI		
Pak et al. (2014)	ASCs	32-year-old female	Meniscal tear	Percutaneous injection of platelet-rich plasma, calcium chloride, and autologous ASCs	VAS score	3 months	Symptoms were improved
					MRI		Repeated MRI showed almost complete disappearance of the torn meniscus
					ROM		—
					Functional rating index		—

Many studies have reported the use of stem-cell-based tissue engineering for the reconstruction of meniscus defects or overall transplantation, the latter of which is very rare in clinical patients. Both intra-articular injection of MSCs and tissue engineering for meniscus injury repair show great clinical prospects. Due to the more complex cellular and biomechanical properties of the meniscus compared with cartilage, the number of intra-articular injections and tissue engineering of the meniscus in clinical trials is far less than that of cartilage (Kwon et al., 2019). Some patients begin with an initial minor meniscus tear and are treated with partial or even total meniscectomy, which does not contribute to the growth of articular cartilage and joint function. If an effective treatment can be provided to promote the regeneration of cells and tissues of the torn or damaged meniscus, it may be more meaningful to protect the normal morphology and structure of the meniscus and joint function. Tissue engineering offers great opportunities to regain an intact meniscus without permanent injuries.

Studies on repairing partial defect and meniscus tear with stem cells are still in their initial stages, mainly limited to *in vitro* and animal experiments, and are yet to be verified by repeatable experiments. Besides, the method of stem cells collection also has an important influence on clinical application. Additionally, despite the positive results obtained from many *in vitro* experiments and animal models, human clinical trials are still urgently required due to a lack of evidence to conclude that stem cells can form long-lasting tissues similar to the native human menisci. Even BMSC, the most thoroughly studied stem cell source, requires further clinical experiments and long-term follow-ups. The tissue engineering of MSCs to repair damaged menisci is promising and might play an indispensable role.

Currently, experiments yield outcomes in animal models for a certain period and it is unknown whether the induced differentiated tissue can bear the function of the meniscus for a long time. The physical and chemical conditions under which stem cells are cultured are yet to be standardized. For example, on the choice of the carrier of cells, the scaffold, a consensus is yet to be reached. Different scaffold material choices and pretreatment methods emerge every year. PRP is a concentrated suspension of multiple growth factors. Poly-lactic co-glycolic acid (PLGA) network scaffolds pretreated with PRP tend to have more adhesion of chondrocytes and the same number of cells required to repair the meniscus can be achieved by seeding fewer cells (Kwak et al., 2017). Single-rotation PRP preparations may be the most advantageous for intra-articular application; however, dual-rotation PRP systems are to be carefully considered before their clinical application (Kisiday et al., 2012). As a replacement for PRP, Platelet-rich fibrin, an autologous fibrin matrix containing abundant platelet and leukocyte cytokines, has emerged. Its advantages over PRP include ease of preparation/application, low cost, and absence of biochemical modifications.

The preparation of biomimetic scaffolds by decellularization of various tissues is a new and developing field in tissue engineering. Some approaches have entered the clinical stage, such as acellular bone removal and cardiac valvular ligaments (Rana et al., 2017). Mesenchymal stem cells can be considered a hopeful candidate for acellular cartilage repair due to their availability, low donor site incidences, differentiation flexibility, immunoregulatory ability, low antigenicity, and nutritional effects. According to the review by Huang et al. (2017), the use of chemotaxis functionalized scaffolds is promising for increasing cell recruitment, increasing cell penetration into the scaffold, and achieving uniform cell distribution. Furthermore, co-culture with mesenchymal stem cells and differentiated cell populations can increase and stabilize their chondrogenic differentiation abilities.

The use of scaffolds also has disadvantages, such as toxic degradation products, material-induced inflammation, and material purity. A self-assembly scaffoldless approach has come into play since 2007. Aufderheide and Athanasiou (2007) co-cultured high density of meniscus FCs and ACs in different ratios on agarose molds, which could prevent cells from attaching to the matrix, and thereby guiding the formation of new cartilage by forcing cells to attach to other cells or the newly synthesized ECM. Other studies also supported the feasibility of a scaffoldless approach during the early years (Hoben et al., 2007; Gunja and Athanasiou, 2009). However, with the introduction of 3D printing, many of the drawbacks have been solved and the focus has shifted to finding optimal scaffold materials and conditions. Unlike scaffolds, culture media are necessary for cell experiments and have been well adapted for different conditions. Fetal bovine serum (FBS) has long been used in cell culture to enhance the growth of ACs and FCs. Gunja and Athanasiou (2009) tried to use a serum-free medium added with insulin-transferrin-Selenium and dexamethasone, which showed that serum deficiency did not impede ECM production or reduce cell adherence to PLLA constructs. Moreover, since it is not an animal product, an immune reaction is reduced and there is no variation common in FBS, which is of great clinical significance. In contrast, Hoben and Athanasiou (2008) reported that the FBS medium is better in overall matrix production and cell proliferation. Therefore, FBS may be regarded as a biochemical tool to increase Collagen II rather than a necessary culture medium component.

No matter how the materials and cells are selected, the common dilemma is that the mechanical properties are not similar to the native properties even after culturing for a long time. Baker et al. (2011) reported that dynamic tensile loading could regulate mesenchymal stem cell transcriptional behavior, stimulate ECM deposition, and enhance the structure’s mechanical properties. Similarly, Yuan et al. (2014) optimized the cell parameters of pulsed direct current stimulation at the microscale and applied it to the repair of full-thickness defects in the graft at the macro scale, which proved that electrical stimulations promoted MCs migration and integrated tissue repair. Particularly, ET-1 is an angiogenic factor that stimulates internal and external MC migration at a microscopic scale and internal and external explant’s integration and repair at a macroscopic scale (Yuan et al., 2015).

In the future, whether through scaffold material selection, cytokine incorporation, or physical or electrochemical stimulation, the common goal is to make the mechanical properties of the regenerated menisci infinitely similar to those of the native ones. Other concerns on the stem cells such as genetic instability, spontaneous transformation, and carcinogenicity have also been raised, considering the multipotent potential and immunosuppressive properties of specific stem cells. Therefore, relevant growth-promoting and supportive therapy after regenerative meniscus implantation is also essential to considerate. To conclude, emerging stem cell therapies provide more effective treatment algorithms for meniscus connective tissue conditions with the goal of joint preservation and more high-quality evidence from all stakeholders involved in stem cell therapy communication is necessary to refine the role of stem cells for connective tissue regeneration with the goal of successful joint preservation.

## References

[B1] Abpeikar Z. Javdani M. Mirzaei S. A. Alizadeh A. Moradi L. Soleimannejad M. (2021). Macroporous Scaffold Surface Modified with Biological Macromolecules and Piroxicam-Loaded Gelatin Nanofibers toward Meniscus Cartilage Repair. Int. J. Biol. Macromolecules 183, 1327–1345. 10.1016/j.ijbiomac.2021.04.151 33932422

[B2] Achatz F. Kujat R. Pfeifer C. Koch M. Nerlich M. Angele P. (2016). *In Vitro* Testing of Scaffolds for Mesenchymal Stem Cell-Based Meniscus Tissue Engineering-Introducing a New Biocompatibility Scoring System. Materials 9 (4), 276. 10.3390/ma9040276 PMC5502969 28773399

[B3] Adesida A. B. Grady L. M. Khan W. S. Millward-Sadler S. J. Salter D. M. Hardingham T. E. (2007). Human Meniscus Cells Express Hypoxia Inducible Factor-1α and Increased SOX9 in Response to Low Oxygen Tension in Cell Aggregate Culture. Arthritis Res. Ther. 9 (4), R69. 10.1186/ar2267 17640365 PMC2206369

[B4] Arora D. Robey P. G. (2022). Recent Updates on the Biological Basis of Heterogeneity in Bone Marrow Stromal Cells/skeletal Stem Cells. Biomater. Transl 3 (1), 3–16. 10.12336/biomatertransl.2022.01.002 PMC9255791 35837340

[B5] Aufderheide A. C. Athanasiou K. A. (2007). Assessment of a Bovine Co-culture, Scaffold-free Method for Growing Meniscus-Shaped Constructs. Tissue Eng. 13 (9), 2195–2205. 10.1089/ten.2006.0291 17630876

[B6] Baboolal T. G. Khalil-Khan A. Theodorides A. A. Wall O. Jones E. McGonagle D. (2018). A Novel Arthroscopic Technique for Intraoperative Mobilization of Synovial Mesenchymal Stem Cells. Am. J. Sports Med. 46 (14), 3532–3540. 10.1177/0363546518803757 30419170 PMC6282154

[B7] Baek J. Sovani S. Choi W. Jin S. Grogan S. P. D'Lima D. D. (2018). Meniscal Tissue Engineering Using Aligned Collagen Fibrous Scaffolds: Comparison of Different Human Cell Sources. Tissue Eng. A 24 (1-2), 81–93. 10.1089/ten.TEA.2016.0205 PMC5770095 28463545

[B8] Baker B. M. Shah R. P. Huang A. H. Mauck R. L. (2011). Dynamic Tensile Loading Improves the Functional Properties of Mesenchymal Stem Cell-Laden Nanofiber-Based Fibrocartilage. Tissue Eng. Part A 17 (9-10), 1445–1455. 10.1089/ten.tea.2010.0535 21247342 PMC3079166

[B9] Caminal M. Fonseca C. Peris D. Moll X. Rabanal R. M. Barrachina J. (2014). Use of a Chronic Model of Articular Cartilage and Meniscal Injury for the Assessment of Long-Term Effects after Autologous Mesenchymal Stromal Cell Treatment in Sheep. New Biotechnol. 31 (5), 492–498. 10.1016/j.nbt.2014.07.004 25063342

[B10] Caplan A. I. (2017). Mesenchymal Stem Cells: Time to Change the Name!. Stem Cell Translational Med. 6 (6), 1445–1451. 10.1002/sctm.17-0051 PMC5689741 28452204

[B11] Cengiz I. F. Maia F. R. da Silva Morais A. Silva-Correia J. Pereira H. Canadas R. F. (2020). Entrapped in Cage (EiC) Scaffolds of 3D-Printed Polycaprolactone and Porous Silk Fibroin for Meniscus Tissue Engineering. Biofabrication 12 (2), 025028. 10.1088/1758-5090/ab779f 32069441

[B12] Centeno C. J. Busse D. Kisiday J. Keohan C. Freeman M. Karli D. (2008). Increased Knee Cartilage Volume in Degenerative Joint Disease Using Percutaneously Implanted, Autologous Mesenchymal Stem Cells. Pain Physician 11 (3), 343–353. 18523506

[B13] Cui X. Hasegawa A. Lotz M. D'Lima D. (2012). Structured Three-Dimensional Co-culture of Mesenchymal Stem Cells with Meniscus Cells Promotes Meniscal Phenotype without Hypertrophy. Biotechnol. Bioeng. 109 (9), 2369–2380. 10.1002/bit.24495 22422555 PMC3391334

[B14] Dai T. y. Pan Z. y. Yin F. (2021). *In Vivo* Studies of Mesenchymal Stem Cells in the Treatment of Meniscus Injury. Orthop. Surg. 13 (8), 2185–2195. 10.1111/os.13002 34747566 PMC8654668

[B15] De Bari C. Dell'Accio F. Karystinou A. Guillot P. V. Fisk N. M. Jones E. A. (2008). A Biomarker-Based Mathematical Model to Predict Bone-Forming Potency of Human Synovial and Periosteal Mesenchymal Stem Cells. Arthritis Rheum. 58 (1), 240–250. 10.1002/art.23143 18163504

[B16] De Luca M. Aiuti A. Cossu G. Parmar M. Pellegrini G. Robey P. G. (2019). Advances in Stem Cell Research and Therapeutic Development. Nat. Cel Biol 21 (7), 801–811. 10.1038/s41556-019-0344-z 31209293

[B17] Desando G. Giavaresi G. Cavallo C. Bartolotti I. Sartoni F. Nicoli Aldini N. (2016). Autologous Bone Marrow Concentrate in a Sheep Model of Osteoarthritis: New Perspectives for Cartilage and Meniscus Repair. Tissue Eng. C: Methods 22 (6), 608–619. 10.1089/ten.TEC.2016.0033 27151837

[B18] Filardo G. Petretta M. Cavallo C. Roseti L. Durante S. Albisinni U. (2019). Patient-specific Meniscus Prototype Based on 3D Bioprinting of Human Cell-Laden Scaffold. Bone Jt. Res. 8 (2), 101–106. 10.1302/2046-3758.82.BJR-2018-0134.R1 PMC6397325 30915216

[B19] Fox A. J. S. Bedi A. Rodeo S. A. (2012). The Basic Science of Human Knee Menisci. Sports health 4 (4), 340–351. 10.1177/1941738111429419 23016106 PMC3435920

[B20] Gamer L. W. Shi R. R. Gendelman A. Mathewson D. Gamer J. Rosen V. (2017). Identification and Characterization of Adult Mouse Meniscus Stem/progenitor Cells. Connect. Tissue Res. 58 (3-4), 238–245. 10.1080/03008207.2016.1271797 28005443

[B21] González-Fernández M. L. Pérez-Castrillo S. Sánchez-Lázaro J. A. Prieto-Fernández J. G. López-González M. E. Lobato-Pérez S. (2016). Assessment of Regeneration in Meniscal Lesions by Use of Mesenchymal Stem Cells Derived from Equine Bone Marrow and Adipose Tissue. Am. J. Vet. Res. 77 (7), 779–788. 10.2460/ajvr.77.7.779 27347833

[B22] Gunja N. J. Athanasiou K. A. (2009). Effects of Co-cultures of Meniscus Cells and Articular Chondrocytes on PLLA Scaffolds. Biotechnol. Bioeng. 103 (4), 808–816. 10.1002/bit.22301 19274749

[B23] Hagmeijer M. Vonk L. A. Vonk L. Fenu M. van Keep Y. Krych A. (2019). Meniscus Regeneration Combining Meniscus and Mesenchymal Stromal Cells in a Degradable Meniscus Implant: an *In Vitro* Study. eCM 38, 51–62. 10.22203/eCM.v038a05 31402442

[B24] He S. Ruan D. Chen Y. Ran J. Chen X. Yin Z. (2019). Characterization and Comparison of Postnatal Rat Meniscus Stem Cells at Different Developmental Stages. Stem Cell Transl Med 8 (12), 1318–1329. 10.1002/sctm.19-0125 PMC6877772 31638337

[B25] Hoben G. M. Athanasiou K. A. (2008). Creating a Spectrum of Fibrocartilages through Different Cell Sources and Biochemical Stimuli. Biotechnol. Bioeng. 100 (3), 587–598. 10.1002/bit.21768 18078296

[B26] Hoben G. M. Hu J. C. James R. A. Athanasiou K. A. (2007). Self-assembly of Fibrochondrocytes and Chondrocytes for Tissue Engineering of the Knee Meniscus. Tissue Eng. 13 (5), 939–946. 10.1089/ten.2006.0116 17484700

[B27] Hoben G. M. Willard V. P. Athanasiou K. A. (2009). Fibrochondrogenesis of hESCs: Growth Factor Combinations and Cocultures. Stem Cell Development 18 (2), 283–292. 10.1089/scd.2008.0024 PMC3132948 18454697

[B28] Hoberg M. Schmidt E. L. Tuerk M. Stark V. Aicher W. K. Rudert M. (2009). Induction of Endostatin Expression in Meniscal Fibrochondrocytes by Co-culture with Endothelial Cells. Arch. Orthop. Trauma Surg. 129 (8), 1137–1143. 10.1007/s00402-008-0766-8 18839188

[B29] Horie M. Choi H. Lee R. H. Reger R. L. Ylostalo J. Muneta T. (2012). Intra-articular Injection of Human Mesenchymal Stem Cells (MSCs) Promote Rat Meniscal Regeneration by Being Activated to Express Indian Hedgehog that Enhances Expression of Type II Collagen. Osteoarthritis and Cartilage 20 (10), 1197–1207. 10.1016/j.joca.2012.06.002 22750747 PMC3788634

[B30] Horie M. Sekiya I. Muneta T. Ichinose S. Matsumoto K. Saito H. (2009). Intra-articular Injected Synovial Stem Cells Differentiate into Meniscal Cells Directly and Promote Meniscal Regeneration without Mobilization to Distant Organs in Rat Massive Meniscal Defect. Stem Cells 27 (4), 878–887. 10.1634/stemcells.2008-0616 19350690

[B31] Hu D. Gu X. Si W. Qin W. Jiao J. Hao Y. (2019). Biosynthesis of Silver Nanoparticles Using Bauhinia Acuminate Flower Extract and Their Effect to Promote Osteogenesis of MSCs and Improve Meniscus Injury Healing. J. Photochem. Photobiol. B: Biol. 197, 111536. 10.1016/j.jphotobiol.2019.111536 31326846

[B32] Huang H. Wang S. Gui J. Shen H. (2016). A Study to Identify and Characterize the Stem/progenitor Cell in Rabbit Meniscus. Cytotechnology 68 (5), 2083–2103. 10.1007/s10616-016-9949-2 26820973 PMC5023581

[B33] Huang Z. Godkin O. Schulze-Tanzil G. (2017). The Challenge in Using Mesenchymal Stromal Cells for Recellularization of Decellularized Cartilage. Stem Cel Rev Rep 13 (1), 50–67. 10.1007/s12015-016-9699-8 27826794

[B34] Jacob G. Shimomura K. Krych A. J. Nakamura N. (2019). The Meniscus Tear: A Review of Stem Cell Therapies. Cells 9 (1), 92. 10.3390/cells9010092 PMC7016630 31905968

[B35] Jayasuriya C. T. Twomey-Kozak J. Newberry J. Desai S. Feltman P. Franco J. R. (2019). Human Cartilage-Derived Progenitors Resist Terminal Differentiation and Require CXCR4 Activation to Successfully Bridge Meniscus Tissue Tears. Stem Cells 37 (1), 102–114. 10.1002/stem.2923 30358021 PMC6312732

[B36] Katagiri H. Muneta T. Tsuji K. Horie M. Koga H. Ozeki N. (2013). Transplantation of Aggregates of Synovial Mesenchymal Stem Cells Regenerates Meniscus More Effectively in a Rat Massive Meniscal Defect. Biochem. Biophysical Res. Commun. 435 (4), 603–609. 10.1016/j.bbrc.2013.05.026 23685144

[B37] Kisiday J. D. McIlwraith C. W. Rodkey W. G. Frisbie D. D. Steadman J. R. (2012). Effects of Platelet-Rich Plasma Composition on Anabolic and Catabolic Activities in Equine Cartilage and Meniscal Explants. Cartilage 3 (3), 245–254. 10.1177/1947603511433181 26069637 PMC4297115

[B38] Koh R. H. Jin Y. Kang B.-J. Hwang N. S. (2017). Chondrogenically Primed Tonsil-Derived Mesenchymal Stem Cells Encapsulated in Riboflavin-Induced Photocrosslinking Collagen-Hyaluronic Acid Hydrogel for Meniscus Tissue Repairs. Acta Biomater. 53, 318–328. 10.1016/j.actbio.2017.01.081 28161573

[B39] Kondo S. Muneta T. Nakagawa Y. Koga H. Watanabe T. Tsuji K. (2017). Transplantation of Autologous Synovial Mesenchymal Stem Cells Promotes Meniscus Regeneration in Aged Primates. J. Orthop. Res. 35 (6), 1274–1282. 10.1002/jor.23211 26916126

[B40] Kremer A. Ribitsch I. Reboredo J. Dürr J. Egerbacher M. Jenner F. (2017). Three-Dimensional Coculture of Meniscal Cells and Mesenchymal Stem Cells in Collagen Type I Hydrogel on a Small Intestinal Matrix-A Pilot Study toward Equine Meniscus Tissue Engineering. Tissue Eng. Part A 23 (9-10), 390–402. 10.1089/ten.TEA.2016.0317 28095754

[B41] Kwak H. S. Nam J. Lee J.-h. Kim H. J. Yoo J. J. (2017). Meniscal Repairin Vivousing Human Chondrocyte-Seeded PLGA Mesh Scaffold Pretreated with Platelet-Rich Plasma. J. Tissue Eng. Regen. Med. 11 (2), 471–480. 10.1002/term.1938 24945790

[B42] Kwon H. Brown W. E. Lee C. A. Wang D. Paschos N. Hu J. C. (2019). Surgical and Tissue Engineering Strategies for Articular Cartilage and Meniscus Repair. Nat. Rev. Rheumatol. 15 (9), 550–570. 10.1038/s41584-019-0255-1 31296933 PMC7192556

[B43] Li C. Hu X. Meng Q. Zhang X. Zhu J. Dai L. (2017). The Potential of Using Semitendinosus Tendon as Autograft in Rabbit Meniscus Reconstruction. Sci. Rep. 7 (1), 7033. 10.1038/s41598-017-07166-z 28765605 PMC5539314

[B44] Li H. Liao Z. Yang Z. Gao C. Fu L. Li P. (2021). 3D Printed Poly(ε-Caprolactone)/Meniscus Extracellular Matrix Composite Scaffold Functionalized with Kartogenin-Releasing PLGA Microspheres for Meniscus Tissue Engineering. Front. Bioeng. Biotechnol. 9, 662381. 10.3389/fbioe.2021.662381 33996783 PMC8119888

[B45] Li Z. Wu N. Cheng J. Sun M. Yang P. Zhao F. (2020). Biomechanically, Structurally and Functionally Meticulously Tailored Polycaprolactone/silk Fibroin Scaffold for Meniscus Regeneration. Theranostics 10 (11), 5090–5106. 10.7150/thno.44270 32308770 PMC7163455

[B46] Liang Y. Idrees E. Szojka A. R. A. Andrews S. H. J. Kunze M. Mulet-Sierra A. (2018). Chondrogenic Differentiation of Synovial Fluid Mesenchymal Stem Cells on Human Meniscus-Derived Decellularized Matrix Requires Exogenous Growth Factors. Acta Biomater. 80, 131–143. 10.1016/j.actbio.2018.09.038 30267878

[B47] Liu F. Xu H. Huang H. (2019). A Novel Kartogenin-Platelet-Rich Plasma Gel Enhances Chondrogenesis of Bone Marrow Mesenchymal Stem Cells *In Vitro* and Promotes Wounded Meniscus Healing *In Vivo* . Stem Cel Res Ther 10 (1), 201. 10.1186/s13287-019-1314-x PMC6615105 31287023

[B48] Matsukura Y. Muneta T. Tsuji K. Koga H. Sekiya I. (2014). Mesenchymal Stem Cells in Synovial Fluid Increase after Meniscus Injury. Clin. Orthop. Relat. Res. 472 (5), 1357–1364. 10.1007/s11999-013-3418-4 24338094 PMC3971249

[B49] Matthies N.-F. Mulet-Sierra A. Jomha N. M. Adesida A. B. (2013). Matrix Formation Is Enhanced in Co-cultures of Human Meniscus Cells with Bone Marrow Stromal Cells. J. Tissue Eng. Regen. Med. 7 (12), 965–973. 10.1002/term.1489 22473741

[B50] McCorry M. C. Bonassar L. J. (2017). Fiber Development and Matrix Production in Tissue-Engineered Menisci Using Bovine Mesenchymal Stem Cells and Fibrochondrocytes. Connect. Tissue Res. 58 (3-4), 329–341. 10.1080/03008207.2016.1267152 27925474 PMC5709035

[B51] McCorry M. C. Puetzer J. L. Bonassar L. J. (2016). Characterization of Mesenchymal Stem Cells and Fibrochondrocytes in Three-Dimensional Co-culture: Analysis of Cell Shape, Matrix Production, and Mechanical Performance. Stem Cel Res Ther 7, 39. 10.1186/s13287-016-0301-8 PMC4789279 26971202

[B52] Meier E. M. Wu B. Siddiqui A. Tepper D. G. Longaker M. T. Lam M. T. (2016). Mechanical Stimulation Increases Knee Meniscus Gene RNA-Level Expression in Adipose-Derived Stromal Cells. Plast. Reconstr. Surg. - Glob. Open 4 (9), e864. 10.1097/GOX.0000000000000854 27757329 PMC5054995

[B53] Mizuno M. Katano H. Otabe K. Komori K. Kohno Y. Fujii S. (2017). Complete Human Serum Maintains Viability and Chondrogenic Potential of Human Synovial Stem Cells: Suitable Conditions for Transplantation. Stem Cel Res Ther 8 (1), 144. 10.1186/s13287-017-0596-0 PMC5470274 28610596

[B54] Moradi L. Vasei M. Dehghan M. M. Majidi M. Farzad Mohajeri S. Bonakdar S. (2017). Regeneration of Meniscus Tissue Using Adipose Mesenchymal Stem Cells-Chondrocytes Co-culture on a Hybrid Scaffold: *In Vivo* Study. Biomaterials 126, 18–30. 10.1016/j.biomaterials.2017.02.022 28242519

[B55] Muhammad H. Schminke B. Bode C. Roth M. Albert J. von der Heyde S. (2014). Human Migratory Meniscus Progenitor Cells Are Controlled via the TGF-β Pathway. Stem Cel Rep. 3 (5), 789–803. 10.1016/j.stemcr.2014.08.010 PMC4235742 25418724

[B56] Nakagawa Y. Muneta T. Kondo S. Mizuno M. Takakuda K. Ichinose S. (2015). Synovial Mesenchymal Stem Cells Promote Healing after Meniscal Repair in Microminipigs. Osteoarthritis and Cartilage 23 (6), 1007–1017. 10.1016/j.joca.2015.02.008 25683149

[B57] Niu W. Guo W. Han S. Zhu Y. Liu S. Guo Q. (20162016). Cell-Based Strategies for Meniscus Tissue Engineering. Stem Cell Int. 2016, 1–10. 10.1155/2016/4717184 PMC4871968 27274735

[B58] Nordberg R. C. Charoenpanich A. Vaughn C. E. Griffith E. H. Fisher M. B. Cole J. H. (2016). Enhanced Cellular Infiltration of Human Adipose-Derived Stem Cells in Allograft Menisci Using a Needle-Punch Method. J. Orthop. Surg. Res. 11 (1), 132. 10.1186/s13018-016-0467-x 27793202 PMC5084349

[B59] Olivos-Meza A. Pérez Jiménez F. J. Granados-Montiel J. Landa-Solís C. Cortés González S. Jiménez Aroche C. A. (2019). First Clinical Application of Polyurethane Meniscal Scaffolds with Mesenchymal Stem Cells and Assessment of Cartilage Quality with T2 Mapping at 12 Months. Cartilage 13, 197S–207S. 10.1177/1947603519852415 31387368 PMC8808880

[B60] Osawa A. Harner C. D. Gharaibeh B. Matsumoto T. Mifune Y. Kopf S. (2013). The Use of Blood Vessel-Derived Stem Cells for Meniscal Regeneration and Repair. Med. Sci. Sports Exerc. 45 (5), 813–823. 10.1249/MSS.0b013e31827d1e06 23247715 PMC4360900

[B61] Ozeki N. Kohno Y. Kushida Y. Watanabe N. Mizuno M. Katano H. (2021). Synovial Mesenchymal Stem Cells Promote the Meniscus Repair in a Novel Pig Meniscus Injury Model. J. Orthop. Res. 39 (1), 177–183. 10.1002/jor.24846 32886427 PMC7821148

[B62] Pak J. Lee J. H. Lee S. H. (2014). Regenerative Repair of Damaged Meniscus with Autologous Adipose Tissue-Derived Stem Cells. Biomed. Res. Int. 2014, 1–10. 10.1155/2014/436029 PMC3925627 24592390

[B63] Peretti G. M. Caruso E. M. Randolph M. A. Zaleske D. J. (2001). Meniscal Repair Using Engineered Tissue. J. Orthop. Res. 19 (2), 278–285. 10.1016/s0736-0266(00)90010-x 11347702

[B64] Qi Y. Chen G. Feng G. (2016a). Osteoarthritis Prevention and Meniscus Regeneration Induced by Transplantation of Mesenchymal Stem Cell Sheet in a Rat Meniscal Defect Model. Exp. Ther. Med. 12 (1), 95–100. 10.3892/etm.2016.3325 27347022 PMC4906666

[B65] Qi Y. Yang Z. Ding Q. Zhao T. Huang Z. Feng G. (2016b). Targeted Transplantation of Iron Oxide-Labeled, Adipose-Derived Mesenchymal Stem Cells in Promoting Meniscus Regeneration Following a Rabbit Massive Meniscal Defect. Exp. Ther. Med. 11 (2), 458–466. 10.3892/etm.2015.2944 26893631 PMC4734176

[B66] Rana D. Zreiqat H. Benkirane-Jessel N. Ramakrishna S. Ramalingam M. (2017). Development of Decellularized Scaffolds for Stem Cell-Driven Tissue Engineering. J. Tissue Eng. Regen. Med. 11 (4), 942–965. 10.1002/term.2061 26119160

[B67] Rothrauff B. B. Sasaki H. Kihara S. Overholt K. J. Gottardi R. Lin H. (2019). Point-of-Care Procedure for Enhancement of Meniscal Healing in a Goat Model Utilizing Infrapatellar Fat Pad-Derived Stromal Vascular Fraction Cells Seeded in Photocrosslinkable Hydrogel. Am. J. Sports Med. 47 (14), 3396–3405. 10.1177/0363546519880468 31644307

[B68] Sakaguchi Y. Sekiya I. Yagishita K. Muneta T. (2005). Comparison of Human Stem Cells Derived from Various Mesenchymal Tissues: Superiority of Synovium as a Cell Source. Arthritis Rheum. 52 (8), 2521–2529. 10.1002/art.21212 16052568

[B69] Saliken D. J. Mulet-Sierra A. Jomha N. M. Adesida A. B. (2012). Decreased Hypertrophic Differentiation Accompanies Enhanced Matrix Formation in Co-cultures of Outer Meniscus Cells with Bone Marrow Mesenchymal Stromal Cells. Arthritis Res. Ther. 14 (3), R153. 10.1186/ar3889 22726892 PMC3446539

[B70] Sasaki H. Rothrauff B. B. Alexander P. G. Lin H. Gottardi R. Fu F. H. (2018). *In Vitro* Repair of Meniscal Radial Tear with Hydrogels Seeded with Adipose Stem Cells and TGF-Β3. Am. J. Sports Med. 46 (10), 2402–2413. 10.1177/0363546518782973 30001494

[B71] Saulnier N. Viguier E. Perrier-Groult E. Chenu C. Pillet E. Roger T. (2015). Intra-articular Administration of Xenogeneic Neonatal Mesenchymal Stromal Cells Early after Meniscal Injury Down-Regulates Metalloproteinase Gene Expression in Synovium and Prevents Cartilage Degradation in a Rabbit Model of Osteoarthritis. Osteoarthritis and Cartilage 23 (1), 122–133. 10.1016/j.joca.2014.09.007 25219668

[B72] Segawa Y. Muneta T. Makino H. Nimura A. Mochizuki T. Ju Y.-J. (2009). Mesenchymal Stem Cells Derived from Synovium, Meniscus, Anterior Cruciate Ligament, and Articular Chondrocytes Share Similar Gene Expression Profiles. J. Orthop. Res. 27 (4), 435–441. 10.1002/jor.20786 18973232

[B73] Sekiya I. Koga H. Katano H. Mizuno M. Kohno Y. Otabe K. (2021). Second-look Arthroscopy after Meniscus Repair and Synovial Mesenchymal Stem Cell Transplantation to Treat Degenerative Flaps and Radial Tears of the Medial Meniscus: A Case Report. J. Orthopaedic Sci. 1, 1. 10.1016/j.jos.2021.04.015 34120825

[B74] Sekiya I. Koga H. Otabe K. Nakagawa Y. Katano H. Ozeki N. (2019). Additional Use of Synovial Mesenchymal Stem Cell Transplantation Following Surgical Repair of a Complex Degenerative Tear of the Medial Meniscus of the Knee: A Case Report. Cel Transpl. 28 (11), 1445–1454. 10.1177/0963689719863793 PMC6802148 31313604

[B75] Shen W. Chen J. Zhu T. Chen L. Zhang W. Fang Z. (2014). Intra-articular Injection of Human Meniscus Stem/progenitor Cells Promotes Meniscus Regeneration and Ameliorates Osteoarthritis through Stromal Cell-Derived Factor-1/cxcr4-Mediated Homing. Stem Cell Transl Med 3 (3), 387–394. 10.5966/sctm.2012-0170 PMC3952922 24448516

[B76] Shen W. Chen J. Zhu T. Yin Z. Chen X. Chen L. (2013). Osteoarthritis Prevention through Meniscal Regeneration Induced by Intra-articular Injection of Meniscus Stem Cells. Stem Cell Development 22 (14), 2071–2082. 10.1089/scd.2012.0563 PMC3700108 23461527

[B77] Shimomura K. Rothrauff B. B. Hart D. A. Hamamoto S. Kobayashi M. Yoshikawa H. (2019). Enhanced Repair of Meniscal Hoop Structure Injuries Using an Aligned Electrospun Nanofibrous Scaffold Combined with a Mesenchymal Stem Cell-Derived Tissue Engineered Construct. Biomaterials 192, 346–354. 10.1016/j.biomaterials.2018.11.009 30471629

[B78] Shimomura K. Rothrauff B. B. Tuan R. S. (2017). Region-Specific Effect of the Decellularized Meniscus Extracellular Matrix on Mesenchymal Stem Cell-Based Meniscus Tissue Engineering. Am. J. Sports Med. 45 (3), 604–611. 10.1177/0363546516674184 27895039

[B79] Somoza R. A. Welter J. F. Correa D. Caplan A. I. (2014). Chondrogenic Differentiation of Mesenchymal Stem Cells: Challenges and Unfulfilled Expectations. Tissue Eng. B: Rev. 20 (6), 596–608. 10.1089/ten.TEB.2013.0771 PMC4241862 24749845

[B80] Song X. Xie Y. Liu Y. Shao M. Wang W. (2015). Beneficial Effects of Coculturing Synovial Derived Mesenchymal Stem Cells with Meniscus Fibrochondrocytes Are Mediated by Fibroblast Growth Factor 1: Increased Proliferation and Collagen Synthesis. Stem Cell Int. 2015, 1–11. 10.1155/2015/926325 PMC4379431 25852755

[B81] Struijk C. Van Genechten W. Verdonk P. Krych A. J. Dietz A. B. Wijnen A. J. (2021). Human Meniscus Allograft Augmentation by Allogeneic Mesenchymal Stromal/stem Cell Injections. J. Orthopaedic Res. 40, 712–726. 10.1002/jor.25074 PMC8578587 33969529

[B82] Stuckensen K. Schwab A. Knauer M. Muiños-López E. Ehlicke F. Reboredo J. (2018). Tissue Mimicry in Morphology and Composition Promotes Hierarchical Matrix Remodeling of Invading Stem Cells in Osteochondral and Meniscus Scaffolds. Adv. Mater. 30 (28), 1706754. 10.1002/adma.201706754 29847704

[B83] Suzuki S. Mizuno M. Sakamaki Y. Mimata A. Endo K. Kohno Y. (2020). Morphological Changes in Synovial Mesenchymal Stem Cells during Their Adhesion to the Meniscus. Lab. Invest. 100 (7), 916–927. 10.1038/s41374-020-0421-8 32238905

[B84] Takata Y. Nakase J. Shimozaki K. Asai K. Tsuchiya H. (2020). Autologous Adipose-Derived Stem Cell Sheet Has Meniscus Regeneration-Promoting Effects in a Rabbit Model. Arthrosc. J. Arthroscopic Relat. Surg. 36 (10), 2698–2707. 10.1016/j.arthro.2020.06.004 32554078

[B85] Tan Y. Zhang Y. Pei M. (2010). Meniscus Reconstruction through Coculturing Meniscus Cells with Synovium-Derived Stem Cells on Small Intestine Submucosa-A Pilot Study to Engineer Meniscus Tissue Constructs. Tissue Eng. Part A 16 (1), 67–79. 10.1089/ten.TEA.2008.0680 19619075

[B86] Tarafder S. Gulko J. Kim D. Sim K. H. Gutman S. Yang J. (2019). Effect of Dose and Release Rate of CTGF and TGFβ3 on Avascular Meniscus Healing. J. Orthop. Res. 37 (7), 1555–1562. 10.1002/jor.24287 30908692 PMC6601329

[B87] Tarafder S. Gulko J. Sim K. H. Yang J. Cook J. L. Lee C. H. (2018). Engineered Healing of Avascular Meniscus Tears by Stem Cell Recruitment. Sci. Rep. 8 (1), 8150. 10.1038/s41598-018-26545-8 29802356 PMC5970239

[B88] Toratani T. Nakase J. Numata H. Oshima T. Takata Y. Nakayama K. (2017). Scaffold-Free Tissue-Engineered Allogenic Adipose-Derived Stem Cells Promote Meniscus Healing. Arthrosc. J. Arthroscopic Relat. Surg. 33 (2), 346–354. 10.1016/j.arthro.2016.07.015 27670757

[B89] Uzieliene I. Urbonaite G. Tachtamisevaite Z. Mobasheri A. Bernotiene E. (2018). The Potential of Menstrual Blood-Derived Mesenchymal Stem Cells for Cartilage Repair and Regeneration: Novel Aspects. Stem Cell Int. 2018, 1–10. 10.1155/2018/5748126 PMC6304826 30627174

[B90] Vangsness C. T. Jr. Farr J. 2nd Boyd J. Dellaero D. T. Mills C. R. LeRoux-Williams M. (2014). Adult Human Mesenchymal Stem Cells Delivered via Intra-articular Injection to the Knee Following Partial Medial Meniscectomy. J. Bone Jt. Surg Am 96 (2), 90–98. 10.2106/jbjs.M.00058 24430407

[B91] Vonk L. A. Kroeze R. J. Doulabi B. Z. Hoogendoorn R. J. Huang C. Helder M. N. (2010). Caprine Articular, Meniscus and Intervertebral Disc Cartilage: An Integral Analysis of Collagen Network and Chondrocytes. Matrix Biol. 29 (3), 209–218. 10.1016/j.matbio.2009.12.001 20005293

[B92] Watanabe N. Endo K. Komori K. Ozeki N. Mizuno M. Katano H. (2020). Mesenchymal Stem Cells in Synovial Fluid Increase in Knees with Degenerative Meniscus Injury after Arthroscopic Procedures through the Endogenous Effects of CGRP and HGF. Stem Cel Rev Rep 16 (6), 1305–1315. 10.1007/s12015-020-10047-0 32996054

[B93] Whitehouse M. R. Howells N. R. Parry M. C. Austin E. Kafienah W. Brady K. (2017). Repair of Torn Avascular Meniscal Cartilage Using Undifferentiated Autologous Mesenchymal Stem Cells: From *In Vitro* Optimization to a First-In-Human Study. Stem Cell Transl Med 6 (4), 1237–1248. 10.1002/sctm.16-0199 PMC5442845 28186682

[B94] Xie X. Zhu J. Hu X. Dai L. Fu X. Zhang J. (2018). A Co-culture System of Rat Synovial Stem Cells and Meniscus Cells Promotes Cell Proliferation and Differentiation as Compared to Mono-Culture. Sci. Rep. 8 (1), 7693. 10.1038/s41598-018-25709-w 29769537 PMC5955983

[B95] Yamasaki T. Deie M. Shinomiya R. Izuta Y. Yasunaga Y. Yanada S. (2005). Meniscal Regeneration Using Tissue Engineering with a Scaffold Derived from a Rat Meniscus and Mesenchymal Stromal Cells Derived from Rat Bone Marrow. J. Biomed. Mater. Res. 75A (1), 23–30. 10.1002/jbm.a.30369 16049928

[B96] Ying X. Z. Qian J. J. Peng L. Zheng Q. Zhu B. Jin Y. H. (2018). Model Research on Repairing Meniscus Injury in Rabbits Using Bone Marrow Mesenchymal Stem Cells and Silk Fibroin Meniscus Porous Scaffold. Eur. Rev. Med. Pharmacol. Sci. 22 (12), 3689–3693. 10.26355/eurrev_201806_15247 29949141

[B97] Yuan X. Arkonac D. E. Chao P.-h. G. Vunjak-Novakovic G. (2014). Electrical Stimulation Enhances Cell Migration and Integrative Repair in the Meniscus. Sci. Rep. 4, 3674. 10.1038/srep03674 24419206 PMC3891019

[B98] Yuan X. Eng G. M. Arkonac D. E. Chao P.-h. G. Vunjak-Novakovic G. (2015). Endothelial Cells Enhance the Migration of Bovine Meniscus Cells. Arthritis Rheumatol. 67 (1), 182–192. 10.1002/art.38889 25307081 PMC4280351

[B99] Yuan X. Wei Y. Villasante A. Ng J. J. D. Arkonac D. E. Chao P.-h. G. (2017). Stem Cell Delivery in Tissue-specific Hydrogel Enabled Meniscal Repair in an Orthotopic Rat Model. Biomaterials 132, 59–71. 10.1016/j.biomaterials.2017.04.004 28407495 PMC5473162

[B100] Zellner J. Pattappa G. Koch M. Lang S. Weber J. Pfeifer C. G. (2017). Autologous Mesenchymal Stem Cells or Meniscal Cells: what Is the Best Cell Source for Regenerative Meniscus Treatment in an Early Osteoarthritis Situation? Stem Cel Res Ther 8 (1), 225. 10.1186/s13287-017-0678-z PMC5634903 29017608

[B101] Zellner J. Taeger C. D. Schaffer M. Roldan J. C. Loibl M. Mueller M. B. (2014). Are Applied Growth Factors Able to Mimic the Positive Effects of Mesenchymal Stem Cells on the Regeneration of Meniscus in the Avascular Zone? Biomed. Res. Int. 2014, 1–10. 10.1155/2014/537686 PMC4164129 25250325

[B102] Zhang Z.-Z. Jiang D. Wang S.-J. Qi Y.-S. Zhang J.-Y. Yu J.-K. (2015). Potential of Centrifugal Seeding Method in Improving Cells Distribution and Proliferation on Demineralized Cancellous Bone Scaffolds for Tissue-Engineered Meniscus. ACS Appl. Mater. Inter. 7 (28), 15294–15302. 10.1021/acsami.5b03129 26102091

[B103] Zhang Z.-Z. Wang S.-J. Zhang J.-Y. Jiang W.-B. Huang A.-B. Qi Y.-S. (2017). 3D-Printed Poly(ε-Caprolactone) Scaffold Augmented with Mesenchymal Stem Cells for Total Meniscal Substitution: A 12- and 24-Week Animal Study in a Rabbit Model. Am. J. Sports Med. 45 (7), 1497–1511. 10.1177/0363546517691513 28278383

[B104] Zhang Z.-Z. Zhou Y.-F. Li W.-P. Jiang C. Chen Z. Luo H. (2019). Local Administration of Magnesium Promotes Meniscal Healing through Homing of Endogenous Stem Cells: A Proof-Of-Concept Study. Am. J. Sports Med. 47 (4), 954–967. 10.1177/0363546518820076 30786213

[B105] Zhao W. Zou T. Cui H. Lv Y. Gao D. Ruan C. (2020). Parathyroid Hormone (1-34) Promotes the Effects of 3D Printed Scaffold-Seeded Bone Marrow Mesenchymal Stem Cells on Meniscus Regeneration. Stem Cel Res Ther 11 (1), 328. 10.1186/s13287-020-01845-x PMC7394673 32731897

[B106] Zhong G. Yao J. Huang X. Luo Y. Wang M. Han J. (2020). Injectable ECM Hydrogel for Delivery of BMSCs Enabled Full-Thickness Meniscus Repair in an Orthotopic Rat Model. Bioactive Mater. 5 (4), 871–879. 10.1016/j.bioactmat.2020.06.008 PMC7332471 32637750

